# Heterozygous* NPR2* Mutation in Two Family Members with Short Stature and Skeletal Dysplasia

**DOI:** 10.1155/2018/7658496

**Published:** 2018-11-28

**Authors:** Marianne Jacob, Surabhi Menon, Christina Botti, Ian Marshall

**Affiliations:** ^1^Rutgers-Robert Wood Johnson Medical School, Department of Pediatrics, 1 Robert Wood Johnson Place, New Brunswick, NJ 08901, USA; ^2^Rutgers-Robert Wood Johnson Medical School, 675 Hoes Lane W, Piscataway Township, NJ 08854, USA; ^3^Rutgers-Robert Wood Johnson Medical School, Division of Pediatric Genetics, 1 Robert Wood Johnson Place, New Brunswick, NJ 08901, USA; ^4^Rutgers-Robert Wood Johnson Medical School, Division of Pediatric Endocrinology, 1 Robert Wood Johnson Place, New Brunswick, NJ 08901, USA

## Abstract

Endochondral ossification at the level of the growth plate, an essential process involved in longitudinal growth, is regulated by hormonal and local factors including C-type natriuretic peptide and its receptor, natriuretic peptide receptor B. Biallelic loss-of-function mutations in the* NPR2 *gene, which encodes this receptor, cause acromesomelic dysplasia, Maroteaux type (AMDM), a skeletal dysplasia characterized by severe short stature and disproportionate shortening of limbs. Heterozygous* NPR2 *mutations have been reported in patients previously classified with idiopathic short stature (ISS). We report the presentation of a 7-year-old girl and her mother with short stature, both of whom were identified with the same* NPR2 *mutation, and who demonstrated clinical and radiological features consistent with a skeletal dysplasia. We also report the patient's response to recombinant human growth hormone (rhGH) over a 2-year period. We encourage clinicians who evaluate children with ISS to consider genetic testing, particularly when the presentation is associated with features suggestive of a skeletal dysplasia.

## 1. Introduction

Idiopathic short stature (ISS) has classically been defined as a height more than 2 standard deviation scores (SDS) below the mean height for age and sex without evidence of systemic, endocrine, nutritional, or chromosomal abnormalities [1]. With the advent of more sophisticated chromosomal testing techniques, including whole exome sequencing (WES), additional etiologies of ISS are being identified, most notably mutations in aggrecan (*ACAN*) and* NPR2 *genes [2, 3].

The* NPR2* gene is located on chromosome 9p21-p12 and encodes natriuretic peptide receptor B (NPR-B) [4]. NPR-B is the receptor for C-type natriuretic peptide (CNP) [4]. In growth plate chondrocytes, binding of CNP to NPR-B stimulates chondrocyte differentiation and hypertrophy, and an increase in matrix synthesis [5]. This interaction plays an important role in endochondral ossification at the level of the growth plate, an important component of longitudinal bone growth [6].

In humans, biallelic* NPR2 *mutations cause acromesomelic dysplasia, Maroteaux type (AMDM) [7]. AMDM is characterized by severe short stature (< -5 SD for height), disproportionate shortening of limbs mainly affecting the middle and distal segments of the upper and lower limbs, bowing of the forearm, and shortening and widening of the metacarpals and phalanges [8]. Family members of a patient diagnosed with AMDM and noted to be short for the general population were identified as heterozygous carriers of* NPR2* mutations [9]. Subsequent studies identified heterozygous* NPR2* mutations as the cause of short stature in unrelated patients previously classified with ISS [5, 6, 10, 11]. Although additional patients with heterozygous* NPR2* mutations are being identified, phenotypic characterization of these patients is incomplete.

We present a 7-year-old girl whose evaluation for short stature revealed clinical and radiological features suggestive of a skeletal dysplasia. Similar features were also identified in her mother. Genetic testing of both revealed* NPR2 *heterozygosity. We also report the patient's response to recombinant human growth hormone (rhGH).

## 2. Case Presentation

**Figure 1:**
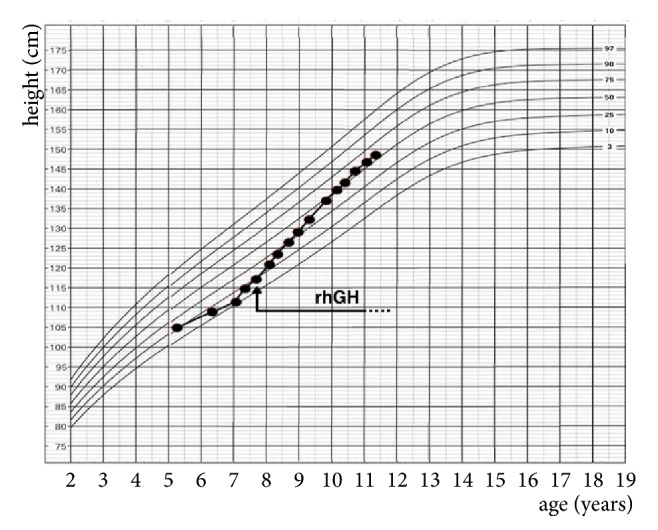
Height growth chart of patient. Recombinant human growth hormone (rhGH) therapy start indicated.

**Figure 2:**
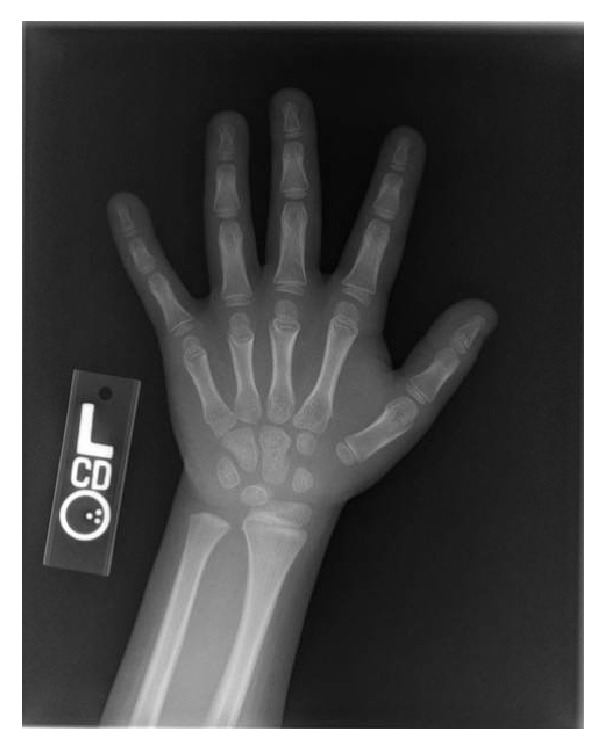
Shortened metacarpals (except the 2nd) of the left hand.

**Figure 3:**
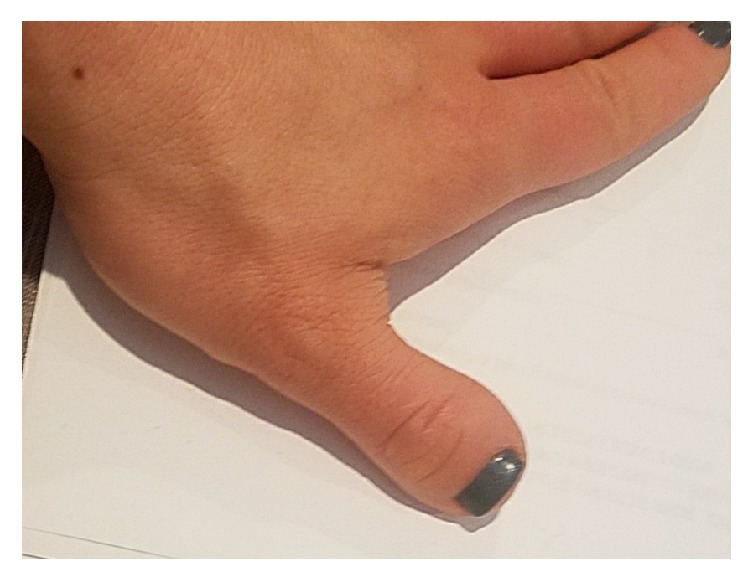
Short and broad thumb of the mother.

The patient presented at age 7 years for evaluation of short stature. Review of her growth chart demonstrated prior stable linear growth below the 1.2nd percentile for age and sex (Figure 1). She was born full term with a birth weight and length appropriate for gestational age. Examination revealed a stocky-appearing girl with a height of 117.5 cm (height SDS -2.5). Her arm span measured 118.4 cm, and arm span to height ratio was normal at 1.01. Her sitting height was 66 cm with a sitting to standing height ratio of 0.56. Her upper and lower segments were measured at 59.7 and 57.8 cm, respectively, giving her a normal upper to lower segment ratio of 1.03. Bilateral 5th finger brachydactyly clinodactyly, bilateral short broad thumbs and first toes, and mild mesomelia of the upper limbs were noted. She did not have clinical evidence of Madelung deformity. Radiological imaging revealed shortened metacarpals of her left hand on a bone age X-ray (Figure 2). She did not have radiological evidence of a Madelung deformity. The mild mesomelia noted clinically was not appreciated on imaging. Her bone age was 7 years 10 months. Her mother was short for her own family with a height of 146 cm (height SDS -2.7) compared to her own midparental target height of 163 cm (height SDS 0.1). Her examination also demonstrated short, broad thumbs (Figure 3) and first toes bilaterally, and mild mesomelia of the upper limbs. Eleven rib pairs were also identified in both the patient and her mother. No other measurements were performed on the mother. Evaluation of the patient's 2 younger siblings, and father (183 cm) did not reveal short stature or any abnormal skeletal features.

Endocrine workup revealed normal GH markers with insulin-like growth factor-1 (IGF-1) of 186 ng/mL (55-218) and insulin-like growth factor binding protein-3 (IGFBP-3) of 3.47 mg/L (2.02-5.52) (Esoterix Endocrinology, Calabasas Hills, CA). Genetic testing showed normal 46,XX karyotype, and negative SHOX mutation analysis (DHPLC, Esoterix Endocrinology, Calabasas Hills, CA). Whole exome sequencing (WES) performed on the patient and both parents at GeneDx (Gaithersburg, MD) revealed heterozygosity for a p.R921X variant [c.2761 C>T (p.Arg921Ter)] in exon 19 of* NPR2* gene in the patient and the mother. The father's testing was negative. This R921X variant has been identified in 1 other patient by Wang et al. [12] with AMDM.

The patient was started on recombinant human growth hormone (rhGH) therapy at age 7.75 years with an increase in her annualized growth velocity from 4.4 cm/yr pre-rhGH to 9.3 cm/yr after year 1 (increase in height SDS from -2.5 to -1.1) and then to 10.5 cm/yr at the end of year 2 (height SDS of 0) (Figure 1). The starting rhGH dose was 0.3 mg/kg/week and was raised to a maximum of 0.34 mg/kg/week over this period of time.

## 3. Discussion

The first report of heterozygous* NPR2* mutations identified in patients with short stature was by Olney et al. in 2005 [9] in which investigation of 39 family members of a patient with AMDM demonstrated mean height SDS of heterozygote* NPR2* carriers to be 1.4 SD below noncarrier family members. Subsequently* NPR2* heterozygosity has been identified in patients evaluated for ISS in studies by Wang et al. [4] in 4% of 192 unrelated ISS patients, Amano et al. [6] in 2% of 101 ISS patients, and Vasques et al. [10] in 6% of 47 patients with ISS.

Nonspecific skeletal abnormalities have been described in the clinical phenotype of individuals with* NPR2 *heterozygosity. However, the frequency of these abnormalities and detailed description of these actual features have not been well documented. Our review of all published reports of ISS patients with* NPR2 *heterozygosity identified a total of 6 articles with a description of skeletal abnormalities in 2 patients – the first patient with shortened metacarpals on a skeletal survey [10], and the second in the obligate heterozygote father of a 10-year-old boy with AMDM, with this father reported to have “small hands” despite normal adult height [12]. Due to the presence of skeletal dysplastic features in Léri-Weill dyschondrosteosis (LWD) which is classically characterized by disproportionate short stature, mesomelia, and Madelung deformity [13], Hisado-Oliva et al. [11] investigated whether* NPR2* mutations could account for a proportion of cases with suspected LWD in whom* SHOX* testing was negative. LWD is typically caused by mutations in the short homeobox (*SHOX*) gene in about 70% of cases [14]. In this study [11],* NPR2* heterozygosity was demonstrated in 7 of 173 patients with suspected LWD and in 2 of an additional cohort of 95 patients with ISS. Although none of the 7 with* NPR2* heterozygosity had the classic Madelung deformity, reported skeletal features included mesomelic limb shortening, cone shaped epiphyses, cubitus valgus, short 5th phalanges and short 4th metacarpals, and/or brachydactyly. Of the 2 ISS patients, one ISS patient had cubitus valgus while phenotypic features were not documented in the other [11].

Variable responses to rhGH treatment have been reported in 8 patients with* NPR2* heterozygosity, with the change in height SDS ranging from – 0.3 to + 1.8 [15]. This variability is likely due to differences in age of initiation, dosing, and/or manipulation of endogenous sex steroids to extend duration of growth [15]. Our patient demonstrated an excellent response to rhGH therapy similar to a recent report by Vasques et al. [15].

## 4. Conclusion

We describe a patient and her mother whose clinical and radiological evaluation for short stature revealed certain skeletal dysplastic features, with genetic testing confirming* NPR2* heterozygosity. Not only does this report add to the phenotypic characterization of* NPR2* heterozygotes, but it also expands the differential diagnosis of what has been historically referred to as idiopathic forms of short stature. We encourage clinicians to consider genetic testing as part of their evaluation of children with ISS, if clinically indicated and particularly if there are features suggestive of a skeletal dysplasia. In these situations, we think the genetic work up is important as it can shorten the time to diagnosis, reduce financial costs of unnecessary additional testing, allow more appropriate genetic counseling of the patient and family, and could provide essential information about the future course including response to any therapies.

Although only 1 patient, we were also able to report the response of our patient to rhGH therapy. Larger studies are obviously necessary to establish the efficacy of rhGH therapy on final height in patients with* NPR2 *heterozygosity.

## References

[1] Cohen L. E. (2014). Idiopathic short stature: A clinical review. *Journal of the American Medical Association*.

[2] Quintos J. B., Guo M. H., Dauber A. (2015). Idiopathic short stature due to novel heterozygous mutation of the aggrecan gene. *Journal of Pediatric Endocrinology and Metabolism*.

[3] Kang M. J. (2017). Novel genetic cause of idiopathic short stature. *Annals of Pediatric Endocrinology & Metabolism*.

[4] Vasques G. A., Arnhold I. J. P., Jorge A. A. L. (2014). Role of the natriuretic peptide system in normal growth and growth disorders. *Hormone Research in Paediatrics*.

[5] Wang S. R., Jacobsen C. M., Carmichael H. (2015). Heterozygous mutations in natriuretic peptide receptor-B (NPR2) gene as a cause of short stature. *Human Mutation*.

[6] Amano N., Mukai T., Ito Y. (2014). Identification and functional characterization of two novel NPR2 mutations in Japanese patients with short stature. *The Journal of Clinical Endocrinology & Metabolism*.

[7] Bartels C. F., Bükülmez H., Padayatti P. (2004). Mutations in the transmembrane natriuretic peptide receptor NPR-B impair skeletal growth and cause acromesomelic dysplasia, type Maroteaux. *American Journal of Human Genetics*.

[8] Kant S. G., Polinkovsky A., Mundlos S. (1998). Acromesomelic dysplasia maroteaux type maps to human chromosome 9. *American Journal of Human Genetics*.

[9] Olney R. C., Bükülmez H., Bartels C. F. (2006). Heterozygous mutations in natriuretic peptide receptor-B (NPR2) are associated with short stature. *The Journal of Clinical Endocrinology & Metabolism*.

[10] Vasques G. A., Amano N., Docko A. J. (2013). Heterozygous mutations in natriuretic peptide receptor-B (NPR2) gene as a cause of short stature in patients initially classified as idiopathic short stature. *The Journal of Clinical Endocrinology & Metabolism*.

[11] Hisado-Oliva A., Garre-Vázquez A. I., Santaolalla-Caballero F. (2015). Heterozygous NPR2 mutations cause disproportionate short stature, similar to Léri-Weill dyschondrosteosis. *The Journal of Clinical Endocrinology & Metabolism*.

[12] Wang W., Song M. H., Miura K. (2016). Acromesomelic dysplasia, type maroteaux caused by novel loss-of-function mutations of the NPR2 gene: Three case reports. *American Journal of Medical Genetics Part A*.

[13] Rosilio M., Huber-Lequesne C., Sapin H., Carel J.-C., Blum W. F., Cormier-Daire V. (2012). Genotypes and phenotypes of children with SHOX deficiency in France. *The Journal of Clinical Endocrinology & Metabolism*.

[14] Fukami M., Seki A., Ogata T. (2016). SHOX Haploinsufficiency as a Cause of Syndromic and Nonsyndromic Short Stature. *Molecular Syndromology*.

[15] Vasques G. A., Hisado-Oliva A., Funari M. F. A. (2017). Long-term response to growth hormone therapy in a patient with short stature caused by a novel heterozygous mutation in NPR2. *Journal of Pediatric Endocrinology and Metabolism*.

